# Work and Retirement Among Women: The Health and Employment After Fifty Study

**DOI:** 10.1093/occmed/kqae035

**Published:** 2024-05-23

**Authors:** G Palermo, S D’Angelo, G Ntani, G Bevilacqua, K Walker-Bone

**Affiliations:** MRC Versus Arthritis Centre for Musculoskeletal Health and Work, University of Southampton, Southampton, UK; MRC Lifecourse Epidemiology Centre, University of Southampton, Southampton, UK; MRC Versus Arthritis Centre for Musculoskeletal Health and Work, University of Southampton, Southampton, UK; MRC Lifecourse Epidemiology Centre, University of Southampton, Southampton, UK; MRC Versus Arthritis Centre for Musculoskeletal Health and Work, University of Southampton, Southampton, UK; MRC Lifecourse Epidemiology Centre, University of Southampton, Southampton, UK; MRC Versus Arthritis Centre for Musculoskeletal Health and Work, University of Southampton, Southampton, UK; MRC Lifecourse Epidemiology Centre, University of Southampton, Southampton, UK; MRC Versus Arthritis Centre for Musculoskeletal Health and Work, University of Southampton, Southampton, UK; MRC Lifecourse Epidemiology Centre, University of Southampton, Southampton, UK; Monash Centre for Occupational and Environmental Health, Monash University, Melbourne, Victoria, Australia

## Abstract

**Background:**

Women increasingly work beyond age 50+ but their occupational health is under-researched.

**Aims:**

To investigate what jobs older contemporary women do, when they exit their jobs and what factors predict job exit.

**Methods:**

Data came from the Health and Employment After Fifty cohort, which recruited women aged 50–64 at baseline in 2013–14 and has followed them up annually collecting: demographic, lifestyle and work information. Exits from employment were mapped longitudinally over five follow-ups. Time-to-first event Cox regression analyses were used to identify risk factors for job exit.

**Results:**

At baseline, 4436 women participated, 64% of whom were working. The proportions of women working at 50–54, 55–60 and over 60 years were 86%, 79% and 38%, respectively. Amongst all women, after adjustment for age, managing comfortably financially and not coping with the mental demands of the job were associated with exit. Risk factors for job exit differed in the age bands: 50–54; 55–59 and >60 years, reflecting socio-economic status, markers of health (musculoskeletal pain and poor self-rated health) and work factors (under-appreciation, job dissatisfaction, temporary/permanent contracts, coping with work’s physical demands).

**Conclusions:**

Factors contributing to exit from work among older women differ by age group, after controlling for perceived financial position, age and mental demands of the job. A number of work characteristics predict job exit and suggest that employers can play an important role in supporting women to continue working until older ages. Identification and treatment of musculoskeletal pain could also enable work amongst older women.

## Teaser

In this 7-year longitudinal study including 4436 women aged 50+, 67% worked at some point. A major driver to remain working was financial hardship whilst financial stability facilitated job exit. Not coping with the mental demands of work and older age are also predictors of exit for all age groups. Employers could facilitate longer working lives for women by increasing satisfaction, appreciation, and reviewing physical and mental demands. Optimizing pathways of care for women with lower limb pain could also facilitate longer working lives.

**Key learning points**

**What is already known about this subject:**

- Increasing numbers of women aged 50+ are in the labour force.
- Women are more likely than men to work part time and in insecure, less well-remunerated jobs.
- It is unclear whether working to older ages will have health benefits for older women, or increase health risks.

**What this study adds:**

- Two-thirds of women aged 50+ years in this population sample worked at some point; at the baseline, the nature of jobs in which older women are working appear to be different by 5-year cohort; younger women are doing more professional, managerial and technical jobs, and less administrative and retail jobs.
- Predictors of job exit among older women are age, perceived difficulty coping with mental demands of work and perceived financial security.
- Leg pain and poor self-rated health are important health risk factors for job exit; working conditions (security, work demands, appreciation, satisfaction) are also important to retain older women working.

**What impact this may have on practice or policy:**

- Employers with female-dominated workforces, for example, health care and education can take measures to maximize retention of older workers through providing secure, satisfying jobs where women feel appreciated, as well as regular appraisal of job demands.
- Lower limb pain, a common problem among older women in the workforce, may jeopardize workability, but healthcare providers have not historically prioritized care pathways for this complaint, although there are evidence-based interventions that can improve outcomes.
- Older women struggling with the physical demands of their jobs are less likely to be in employment; the role of these women in caring needs recognition and support to make it sustainable.

## INTRODUCTION

Employment is generally good for health, providing purpose, status, financial stability and intrinsic reward [1,2]. Over the past 50 years, the proportion of employed women has increased (e.g. 72% of UK women aged 16–64 years were working in 2020 compared with 55% in 1981 and 53% in 1971) [3,4]. Some of this change is demographic, but this group has also seen the largest growth in employment rates [5]. Moreover, in the UK, there have been changes in the age of entitlement to the state pension 2011–18, which particularly affected a cohort of women born after March 1950, who were originally eligible for pension aged 60 years but for whom the eligibility age increased rapidly, and some will only be eligible at 67 years [6]. Certainly, much of the expansion in older women working has occurred since the 2008 global financial crisis, and it is likely that increasing numbers of women return to work in their 40s–60s for financial reasons [5,7,8] and perhaps have little choice of work.

Whilst the benefit of employment for health is accepted generally, less is known about the benefits, or harms, of working to older ages and in particular, among women. In general, female occupational health has been under-researched and, even when studies include male and female workers, gender-specific analyses are rarely reported [9,10]. Men and women differ importantly in terms of the jobs they do, the tasks they perform at work, their anthropometry, metabolism and endocrinology, and they often have different responsibilities and demands outside of work [9]. Also, women are considerably more likely than men to work part time [4] and to undertake work that is poorly remunerated with precarious or zero-hours contracts [5]. Such jobs may offer less in the way of health benefits than more stable, well-remunerated work.

In 2013–14, the Health and Employment After Fifty (HEAF) cohort study was incepted to better understand health and work/retirement decisions among contemporary middle-aged adults [11]. Using data from women in this cohort (born 1949–63 and most affected by the UK pension reforms [6]), we investigated whether there were age-period cohort effects in the rate of exit from work and factors predicting work exit and the extent to which their health facilitated or prevented them from working to older ages.

## METHODS

The HEAF study recruited people aged 50–64 years at baseline using 24 General Practice registers as the sampling frame. The GP practices were from across England, selected among those who contribute data to the Clinical Practice Research Datalink, a national database that collects anonymized patient data. All registered women in participating practices were mailed a baseline questionnaire, patient information sheet and consent form, unless deemed unethical by their GP (e.g. due to terminal illness). Participants who returned the signed consent form and baseline questionnaire to the study team were recruited. Further details about the study methodology are available elsewhere [11]. Participants were sent five follow-up questionnaires between baseline and the fifth follow-up in 2019, enquiring about socio-demographic characteristics, employment status, working conditions, financial circumstances and health. It was decided a priori to investigate the current research questions in three age cohorts: 50–54, 55–59 and 60+ years to facilitate exploration of any differential impact of recent changes in the age of entitlement to state pension.

The following potential risk factors for work exit were investigated: age, marital status, education, housing tenure, financial dependants, informal caregiving, financial circumstances, obesity, alcohol consumption, smoking status, self-rated health (SRH), depression, musculoskeletal pain, type of employment contract, occupation, shift work, job satisfaction, job security, and perceived coping with mental and/or physical demands of the job. Socio-demographic and lifestyle characteristics were treated as fixed baseline covariates, except for ‘managing financially’, marital status and health markers.

The health measures were SRH, which was dichotomized as ‘fair/poor’ versus ‘at least good’ [12]; Centre for Epidemiologic Score—Depression (CES-D), with a score of ≥16 defined as ‘depressed’ [13]; musculoskeletal pain, questionnaire responses about pain lasting at least 1 month in the past year at one or more of the back, arms/shoulders or legs.

At baseline and each follow-up, participants were asked about their current employment status, and any change since the previous questionnaire. If they reported a job change, they were asked for the dates of leaving the previous job and starting a new one. The outcome of these analyses was time until the first job exit for any reason during the five follow-ups.

Initially, a descriptive analysis was carried out, classifying women into categories according to age at baseline and work pathways longitudinally. The work pathways were *Never exited workforce*; *Never worked*; *Worked at baseline and exited once, and did not return to work (*i.e. *retired)*; and *Other pathways*, which included women not working at baseline who started work working at a later time point and women working at baseline who stopped and started work at least once during follow-up.

To explore risk factors associated with job exit, a multiple-record dataset was generated with fixed and time-varying covariates for factors that could change over time. Each row of this dataset represented a time period during which a respondent was ‘at risk’ of a job exit (either: the time between two questionnaires where employment status remained unchanged; the time between a questionnaire and a job exit; or the time between the start of a job and the subsequent questionnaire). Each row of the dataset recorded the status of the respondent at the end of the time period as either: employed or not employed.

We used Cox proportional hazards models to investigate risk factors for time to first job exit, considering remaining in employment and women lost to follow-up as censoring events. Effect estimates were expressed as hazard ratios (HRs) and 95% confidence intervals (95% CIs). Before proceeding to the final mutually adjusted models, we used a forward selection modelling strategy to identify key predictors. The proportional hazards assumption in the final models were tested by incorporating time-varying covariates, which are interactions between predictors and time, using interactions with log(time).

Ethical approval was obtained from the National Health Service Research Ethics Committee North West-Liverpool East (Reference 12/NW/0500) and all participants gave written informed consent to participate.

## RESULTS

**Figure 1.**
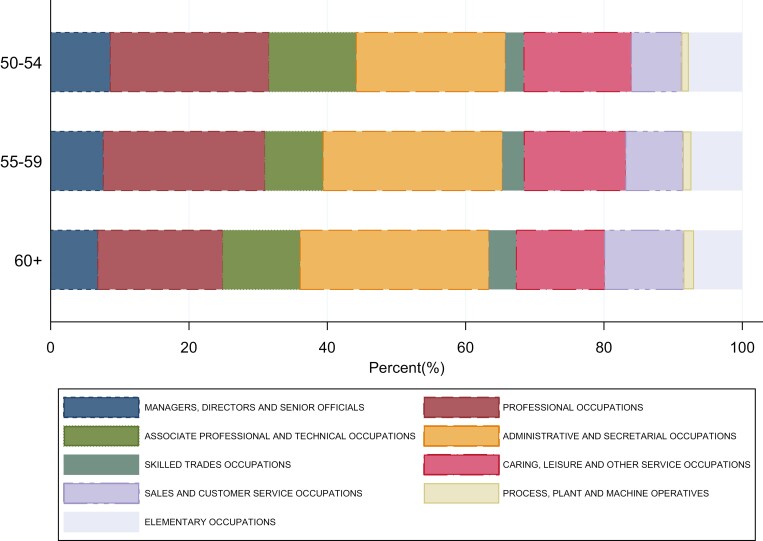
HEAF Women in work (*n* = 2381) at baseline by Occupational Sector and Age groups.

In total, 4436 women participated in HEAF, amongst whom 2950 (67%) worked at some point between baseline and the 5 follow-ups (over almost 7 years), of whom 2831 (64%) were working at baseline, completed at least one follow-up questionnaire and were therefore included in the survival analysis. Eighty-six percent of women aged 50–54 years, 79% of women aged 55–59 and 38% of those aged 60+ years were working at baseline. The types of jobs in which they were working are summarized by Standard Occupational Classification 2010 (SOC-10 [14],) categories (Figure 1). Within this 15-year distribution, larger numbers of younger women were in managerial/professional and technical roles whilst larger numbers of the older women were in administrative/secretarial or retail occupations.

Table 1 summarizes key demographics, socio-economic and health variables for each of the three age categories, reporting, for each variable, the proportion of women who were working versus not working, for example, amongst women aged 60+, the percentage in paid employment was 45% amongst single women as compared with 35% amongst women married/in a partnership. For both age groups 55–59 years (82% versus 76%) and 60+ (51% versus 33%), the percentage of women in employment was higher amongst those not owning their homes as compared with women owning their homes outright. Higher proportions of those who rated their health ‘at least good’ were working in each age band and the same pattern was seen for women reporting no musculoskeletal pain. Women giving more than 20 hours of personal care a week were less likely to be working compared with those giving <20 hours a week.

**Table 1.** Baseline characteristics by work status within each age group—all women at baseline (*n* = 4436)

Baseline, *n* (%)	50–54, 1161 (26)		55–59, 1405 (32)		60+, 1870 (42)	
Status	Not in work, *n* (%)	In work, *n* (%)	Not in work, *n* (%)	In work, *n* (%)	Not in work, *n* (%)	In work, *n* (%)
Participants	158 (14)	1003 (86)	293 (21)	1112 (79)	1154 (62)	716 (38)
Marital status						
Married/civil part/living with partner	101 (13)	663 (87)	213 (23)	724 (77)	843 (65)	458 (35)
Single/widowed/divorced	56 (15)	329 (85)	78 (17)	378 (83)	302 (55)	249 (45)
University degree						
No	122 (14)	725 (86)	209 (20)	843 (80)	903 (61)	578 (39)
Yes	36 (11)	278 (89)	84 (24)	269 (76)	251 (65)	138 (35)
Managing financially						
Living comfortably	39 (12)	277 (88)	103 (24)	327 (76)	504 (68)	234 (32)
Doing alright	40 (10)	348 (90)	68 (13)	436 (87)	368 (56)	284 (44)
Just about getting by/finding it difficult to make	75 (18)	349 (82)	121 (27)	331 (73)	264 (59)	181 (41)
Financial dependents outside household						
No	143 (14)	873 (86)	273 (22)	995 (78)	1093 (62)	667 (38)
Yes	11 (10)	99 (90)	16 (14)	96 (86)	40 (56)	32 (44)
House tenure						
Owned outright	48 (12)	351 (88)	172 (24)	549 (76)	893 (67)	444 (33)
Mortgage/rent (free or not)	107 (15)	620 (85)	119 (18)	544 (82)	246 (49)	253 (51)
Ever smoked regularly						
No	82 (12)	602 (88)	163 (21)	617 (79)	656 (64)	371 (36)
Yes	76 (16)	401 (84)	127 (21)	484 (79)	482 (59)	333 (41)
Alcohol intake per week						
Low/no drinker (≤1 unit per week)	51 (16)	270 (84)	76 (21)	289 (79)	284 (65)	155 (35)
Moderate (2–14 units per week)	62 (10)	559 (90)	138 (18)	633 (82)	595 (59)	410 (41)
Heavy drinker (15+ units per week)	15 (17)	75 (83)	27 (25)	79 (75)	64 (61)	41 (39)
Obese						
<30 (non-obese)	96 (12)	732 (88)	197 (19)	818 (81)	857 (61)	541 (39)
≥30 (obese)	56 (19)	239 (81)	82 (24)	263 (76)	262 (63)	154 (37)
CES-D score						
Less than 16	82 (10)	705 (90)	180 (18)	795 (82)	880 (62)	537 (38)
16 or more (symptoms of depression)	73 (20)	294 (80)	108 (26)	309 (74)	253 (60)	171 (40)
Self-rated health						
At least good	75 (8)	821 (92)	172 (16)	895 (84)	843 (60)	569 (40)
Fair/poor	80 (32)	173 (68)	119 (37)	199 (63)	281 (69)	125 (31)
Pain in the back (past 12 months)						
No	97 (11)	817 (89)	202 (18)	931 (82)	914 (61)	593 (39)
Yes	59 (24)	183 (76)	91 (34)	176 (66)	231 (67)	116 (33)
Pain in the arms/shoulders (past 12 months)						
No	107 (11)	864 (89)	212 (18)	967 (82)	961 (61)	619 (39)
Yes	49 (26)	137 (74)	77 (35)	140 (65)	184 (67)	91 (33)
Pain in the legs (past 12 months)						
No	104 (10)	911 (90)	211 (18)	976 (82)	956 (60)	631 (40)
Yes	52 (37)	88 (63)	81 (39)	129 (61)	189 (71)	79 (29)
Any pain (past 12 months)						
No	80 (10)	739 (90)	174 (17)	839 (83)	816 (61)	529 (39)
Yes	76 (22)	262 (78)	119 (31)	266 (69)	331 (65)	181 (35)
Hours per week giving personal care						
<20 hours	143 (13)	974 (87)	268 (20)	1076 (80)	1085 (61)	691 (39)
20+ hours	15 (34)	29 (66)	25 (41)	36 (59)	69 (73)	25 (27)

During follow-up, 1800 (41%) women worked throughout, 929 working at baseline stopped working at one point and stopped thereafter (‘retired’), 60 women not working at baseline started working, and 161 took ‘other’ pathways, the commonest of which were working, stopped and re-started (*n* = 60); not working at baseline, started and then stopped again (*n* = 55); working at baseline and stopped and started and stopped again during follow-up (*n* = 41). Table 2 describes the characteristics of women within each of the work pathways. The 1800 women who worked throughout were more likely single, reporting struggling financially, not homeowners and depressed. In contrast, 1486 (34%) women who never worked throughout the course of the study were more likely in a partnership; managing comfortably financially; without financial dependants; homeowners; experiencing fair/poor SRH and reporting pain at one or more sites; and providing 20+ hours of personal care. The 929 women working at baseline who exited work once had lower rates of depression and better SRH.

**Table 2.** Comparison of baseline personal and lifestyle characteristics amongst HEAF women, according to their work pathway during follow-up (*n* = 4436)

Baseline	Never exited workforce, *n* (%)	Never worked, *n* (%)	Worked at baseline and exited once, *n* (%)	Other pathways, *n* (%)
Participants	1800 (100)	1486 (100)	929 (100)	221 (100)
Marital status				
Married/civil partnership/living with partner	1141 (63)	1072 (72)	646 (70)	143 (65)
Single/widowed/divorced	640 (36)	403 (27)	273 (29)	76 (34)
University degree				
No	1408 (78)	1154 (78)	669 (72)	149 (67)
Managing financially				
Living comfortably	462 (26)	606 (41)	343 (37)	73 (33)
Doing alright	659 (37)	453 (30)	376 (40)	56 (25)
Just about getting by/finding it difficult	636 (35)	407 (27)	191 (21)	87 (39)
Anyone outside your household financially dependent on you				
No	1606 (89)	1401 (94)	835 (90)	202 (91)
House tenure				
Owned outright	731 (41)	1052 (71)	564 (61)	110 (50)
Mortgage/rent (free or not)	1021 (57)	417 (28)	346 (37)	105 (48)
Ever smoked regularly				
Yes	788 (44)	637 (43)	387 (42)	91 (41)
Alcohol intake per week				
Low/no drinker (≤1 unit per week)	472 (26)	380 (26)	220 (24)	53 (24)
Moderate (2–14 units per week)	987 (55)	728 (49)	549 (59)	133 (60)
Heavy drinker (15+ units per week)	130 (7)	99 (7)	59 (6)	13 (6)
Obese				
BMI ≥ 30 kg/m^2^ (obese)	409 (23)	372 (25)	222 (24)	53 (24)
CES-D score				
16 or more (symptoms of depression)	512 (28)	394 (27)	222 (24)	80 (36)
Self-rated health				
At least good	1456 (81)	1005 (68)	754 (81)	160 (72)
Fair/poor	307 (17)	452 (30)	165 (18)	53 (24)
Pain in the back (past 12 months)				
Yes	295 (16)	354 (24)	160 (17)	47 (21)
Pain in the arms/shoulders (past 12 months)				
Yes	245 (14)	285 (19)	107 (12)	41 (19)
Pain in the legs (past 12 months)				
Yes	183 (10)	306 (21)	101 (11)	28 (13)
Any musculoskeletal pain (past 12 months)				
Yes	454 (25)	488 (33)	227 (24)	66 (30)
Hours per week giving personal care				
20+ hours	57 (3)	98 (7)	30 (3)	14 (6)

The percentages are based on non-missing values that might differ for each variable.

Table 3 describes the work characteristics at baseline by age categories. In the 60+ age group, more women were on temporary contracts or self-employed; more likely to report coping well with the work’s mental demands; feeling appreciated at work; not doing heavy lifting at work and less likely to have been off sick.

**Table 3.** Comparison of baseline work characteristics amongst HEAF women according to their age group (*n* = 2831)

Baseline	50–54, *n* (%)	55–59, *n* (%)	60+, *n* (%)
Participants	1003 (100)	1112 (100)	716 (100)
Employment contract			
Permanent	859 (86)	923 (83)	558 (78)
Temporary/renewable	45 (4)	69 (6)	48 (7)
Self-employed	95 (9)	108 (10)	94 (13)
Occupations SOC2010 (1 digit)			
Professional Occupations	229 (23)	258 (23)	127 (18)
Administrative and Secretarial Occupations	215 (21)	286 (26)	192 (27)
Caring, Leisure and Other Service Occupations	155 (15)	162 (15)	90 (13)
Associate Professional and Technical Occupations	126 (13)	93 (8)	79 (11)
Managers, Directors and Senior Officials	86 (9)	84 (8)	48 (7)
Elementary Occupations	77 (8)	81 (7)	49 (7)
Sales And Customer Service Occupations	72 (7)	91 (8)	80 (11)
Skilled Trades Occupations	27 (3)	35 (3)	28 (4)
Process, Plant and Machine Operatives	11 (1)	14 (1)	11 (2)
Job involves rotating or variable shifts			
Sometimes/rarely	836 (83)	931 (84)	598 (84)
Often	160 (16)	167 (15)	94 (13)
Satisfaction with job as whole			
Satisfied	932 (93)	1028 (92)	668 (93)
Dissatisfied	66 (7)	72 (6)	32 (4)
Thinks job is secure			
Secure	514 (51)	586 (53)	393 (55)
Insecure	485 (48)	514 (46)	307 (43)
Coping with the physical demands of job			
Easily	718 (72)	727 (65)	505 (71)
Just about or worse	278 (28)	372 (33)	196 (27)
Coping with the mental demands of job			
Easily	624 (62)	703 (63)	528 (74)
Just about or worse	375 (37)	397 (36)	171 (24)
Feels appreciated by others at work			
Often/sometimes	893 (89)	1008 (91)	655 (91)
Rarely/never	105 (10)	92 (8)	45 (6)
Job involves heavy lifting			
No	867 (86)	958 (86)	634 (89)
Days off work because of health (past 12 months)			
0 days	516 (51)	540 (49)	398 (56)
1–4 days	316 (32)	327 (29)	183 (26)
5 days or more	167 (17)	238 (21)	124 (17)

The percentages are based on non-missing values that might differ for each variable.

**Figure 2.**
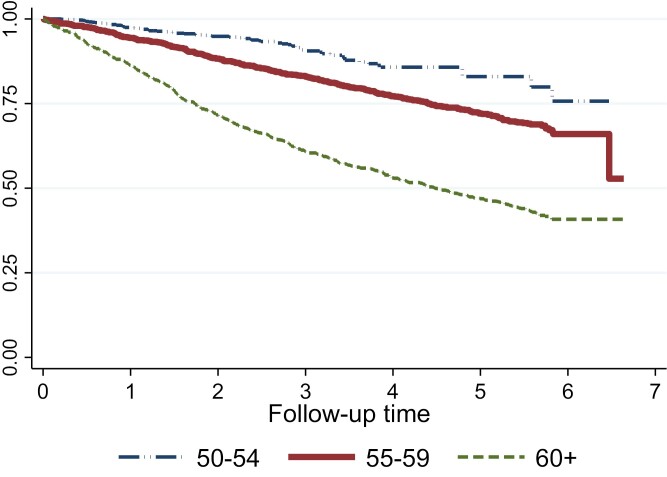
Kaplan–Meier survival estimates for women in work at baseline (*n* = 2831), showing exits from paid work by age categories: 50–55, 56–60 and >60 years.

Figure 2 illustrates survival estimates for each age category, from baseline to the fifth follow-up. Women aged 50–54 were most likely to remain working throughout, followed by those aged 55–59 and 60+. The analysis of mutually adjusted HRs for job loss by age group (Table 4) found that, amongst women of any age: reporting managing financially ‘alright’; not coping with the mental demands of the job and being older were the significant predictors of exit after applying a forward model-building approach. Amongst women aged 50–54, not feeling appreciated at work (HR 2.17, 95% CI 1.19–3.95) and reporting lower limb pain in the past 12 months (HR 2.60, 95% CI 1.41–4.77) were additional predictive factors. Amongst women aged 55–59 years, additional predictors were having temporary (HR 3.88, 95% CI 1.98–7.61) or permanent jobs (HR 2.58, 95% CI 1.44–4.62) (as compared with self-employment); job dissatisfaction (HR 1.52, 95% CI 1.03–2.23); fair/poor SRH (HR 1.72, 95% CI 1.29–2.28); University degree (HR 1.66, 95% CI 1.30–2.11); and working in a job that does not involve heavy lifting (HR 1.85, 95%CI 1.16-2.95). Amongst women aged 60+, temporary (HR 2.17, 95%CI 1.45-3.24) and permanent (HR 1.80; 95% CI 1.34–2.42) employment (as compared with self-employment) were risk factors, as was perceived difficulty in coping with physical demands at work (HR 1.49; 95% CI 1.21–1.85).

**Table 4.** Association between risk factors and job exit between baseline and fifth follow-up amongst 2831 HEAF women in work at baseline, by 5-year age groups.

Risk factor	Age groups (years): HR (95% CI)		
	50–54	55–59	60+
Age, years	1.48 (1.13, 1.94)	1.28 (1.18, 1.39)	1.11 (1.07, 1.15)
Managing financially			
Living comfortably	2.24 (1.23, 4.10)	2.65 (1.88, 3.74)	1.60 (1.22, 2.10)
Doing alright	1.11 (0.58, 2.11)	1.46 (1.02, 2.07)	1.52 (1.17, 1.97)
Just about getting by/finding it difficult	Ref	Ref	Ref
Coping with mental demands of the job			
Easily	Ref	Ref	Ref
Just about or worse	2.27 (1.40, 3.66)	1.65 (1.29, 2.11)	1.28 (1.03, 1.59)
Type of contract			
Permanent	–	2.58 (1.44, 4.62)	1.80 (1.34, 2.42)
Temporary/renewable		3.88 (1.98, 7.61)	2.17 (1.45, 3.24)
Self-employed		Ref	Ref
Feel appreciated by others at work			
Often/sometimes	Ref	–	
Rarely/never	2.17 (1.19, 3.95)		
Pain in the legs (past 12 months)			
No	Ref	–	–
Yes	2.60 (1.41, 4.77)		
Coping with the physical demands of job			
Easily	–	–	Ref
Just about or worse			1.49 (1.21, 1.85)
Self-rated health	–		
At least good	–	Ref	–
Fair/poor		1.72 (1.29, 2.28)	
University degree	–		–
No	–	Ref	–
Yes		1.66 (1.30, 2.11)	
Satisfaction with job as whole			
Satisfied	–	Ref	–
Dissatisfied		1.52 (1.03, 2.23)	
Job involves heavy lifting	–		–
No	–	1.85 (1.16, 2.95)	–
Yes		Ref	

## DISCUSSION

In this pre-pandemic study including 4436 women most affected by UK pension reforms over 7 years, 34% never worked, 41% worked throughout, 21% were working at baseline and had one job exit, and 5% were either not working at baseline and joined the workforce subsequently, or exited and re-entered the workforce several times. At baseline, 86% of women aged 50–54 years, 79% of women aged 55–59 years and 38% of women aged 60+ years were working. Whilst they worked across all sectors, larger numbers of the youngest women were working in managerial/professional and technical roles compared with the older women, who were more commonly in administrative or retail roles. Job insecurity was reported by almost half. As compared with older women not working, working women were more likely to be single, struggling financially, have financial dependants and not own their homes.

Amongst the 2831 women working at baseline, regardless of their age, three common predictors were identified: perception of not coping with work’s mental demands; older age and reporting managing financially ‘alright’. However, we found that different factors predicted workforce exit in the three age groups. Work factors included under-appreciation at work; not being self-employed; job dissatisfaction and perception of not coping with physical demands of work. Important health markers were poor SRH and leg pain over the past 12 months. There were some differences by age: women aged 50–54 years feeling under-appreciated at work were 2.17 times (95% CI 1.17–3.95) more likely to stop working than women feeling appreciated whilst women aged 55–59 years with a university degree were 1.66 times (95% CI 1.30–2.11) more likely to exit paid work than those without.

Health and work are, of course, intertwined. At one extreme, those who are least healthy may be completely unable to work because of their health condition. For example, researchers found that subjective poor health was associated with a significantly increased risk of early retirement (pooled relative risk 1.27, 95% CI 1.17–1.38) and disability pension [15,16], a finding replicated here amongst women aged 55–59 years, (HR 1.72, 95% CI 1.29–2.81). SRH is a very widely used measure, predictive of objective disease [17] and mortality [18] performing more reliably than other, more objective, health metrics [19]. It is likely that, when assessing their personal health, people consider how they are coping and feeling whilst at work, which may explain why it is such an important predictor of work cessation. There is a recognized social gradient in SRH, affected by physical (such as housing), psychosocial and behavioural factors [20]. However, as seen here, good health also influences early retirement, particularly amongst people with better socio-economic situation who perceive that they can afford to retire and enjoy their retirement whilst in good health [21]. Indeed, amongst people working past the traditional age of retirement, good health was a prerequisite [22,23].

We found that leg pain was an important determinant of exit amongst women aged 50–54 years (HR 2.60, 95% CI 1.41–4.77). A common cause of leg pain is osteoarthritis, particularly of the hip and/or knee joints, which is more common among women than men and increases in prevalence with age [24]. However, many people with these complaints do not seek health care [25] and, even for those who do, pathways of care can be unclear or even ineffective [26]. There could be an important role for improved recognition of the potential threat of lower limb pain to workability amongst female workers. Evidence-based cost-effective treatments, including education, diet and exercise, could enable women with leg pain to remain working, which will be particularly important in sectors with a high proportion of women workers, for example, health care [27].

In total, 30% of the work exits reported by women in HEAF were attributed to health whilst 70% were not, a finding consistent with that reported by the Office of National Statistics in 2022 [28]. Our data re-affirm that pre-pandemic, older women were returning to work and remaining in work largely for financial reasons, as has been reported elsewhere [5,7,21]. Socio-economic factors including being single/widowed/divorced, not having a university degree, struggling financially, having financial dependants, not owning their home were all importantly associated with working in women aged 55+ years in HEAF. Whilst retaining older workers in the workforce is thought to be desirable by governments for reducing the old age dependency ratio and pensions costs, our results suggest that it is important that staying in work does not become a long-term imperative for people with the greatest socio-economic disadvantage who cannot afford not to work. Certainly, if these women remain in work instead of, for example, providing informal care [29–31], society may be worse off, particularly if working is at the cost of their own health leading to disablement and requirement for long-term health and social care.

We found important work factors that could be addressed by employers to support women to work to older ages. Women aged 55 and older who were self-employed were more likely to be working, compared to those on temporary or permanent job contracts. Lack of appreciation and job dissatisfaction were importantly associated with the risk of exiting the workforce as has been shown by others [32] and was found by us previously among women in HEAF [33]. This may suggest that appreciation and satisfaction are more important to older female workers than older male workers. There may also be a role for regular appraisal between the older woman worker and their supervisor/manager to review the work demands and consider job modifications that might enable work in the longer term. Job insecurity was common, a factor which is recognized to have long-term negative effects on both health and well-being [34]. Moreover, employees who perceive their jobs to be threatened tend to be less adaptable at work, less productive and show poorer work attitudes [35]. This suggests that there could be benefits to employers and employees in helping women at work to feel secure in their work and supported by the physical and mental demands of their occupational tasks. Unfortunately, women generally tend to have less secure jobs than men and there is evidence that job insecurity is currently considerably increased as a consequence of the COVID-19 pandemic [36]. Financial stability is clearly a determinant of working aged 55+ and it is noteworthy that the widest gender pay gap (>30%) is currently found amongst women aged 55+ years [37].

The results need to be considered alongside some limitations. At baseline, the recruitment rate was approximately 21% and response rates were higher amongst older and wealthier people [11]. That said, a wide geographical spread was represented and every decile of deprivation. Additionally, retention in the HEAF study has been excellent (>69% by follow-up 5), allowing strong comparisons to be made within individuals over time and reducing any potential recall bias. It is important to bear in mind that everybody in the baseline sample has ‘survived’ in work until the age of at least 50 years. Consequently, women who were already too sick to work, or had the option not to work (e.g. for financial reasons), are excluded from making a subsequent work exit. For this reason, the current analyses considered patterns of any type of job exit so that retirement, as well as exits made specifically for health reasons, are visible within these data. Importantly, the data in HEAF are self-reported, which is particularly important in relation to some measures, for example, SRH and ‘coping financially’.

In summary, an increasing proportion of older women are working, often for financial reasons. Aside from age and mental demands of the job, there are important work characteristics that affect exit longitudinally and could be modifiable by employers, for example, appreciation and satisfaction.
